# Maternal Depression and Its Relationship With Cognitive Development in Children Aged 0 to 36 Months: A Systematic Review

**DOI:** 10.62641/aep.2339

**Published:** 2026-10-15

**Authors:** Camila Manríquez Fuenzalida, Diego Henríquez, Alejandra Caqueo-Urízar

**Affiliations:** ^1^Programa de Doctorado en Psicología, Escuela de Psicología y Filosofía, Universidad de Tarapacá, 1000000 Arica, Chile; ^2^Centro de Justicia Educacional (CJE), Pontificia Universidad Católica de Chile, 7820436 Santiago, Chile; ^3^Instituto de Alta Investigación, Universidad de Tarapacá, 1000000 Arica, Chile

**Keywords:** maternal depression, cognitive development, problem-solving, early childhood, systematic review

## Abstract

**Background:**

Maternal depression during the perinatal and postpartum periods constitutes a relevant risk factor for early child development. This systematic review aims to synthesize empirical evidence on the association between maternal depression and child development in children aged 0 to 36 months, considering symptom severity, chronicity, and trajectories, as well as potential relational and contextual mechanisms.

**Methods:**

A systematic review was conducted following Preferred Reporting Items for Systematic reviews and Meta-Analyses (PRISMA) 2020 guidelines. Searches were performed in PsycINFO, SciELO, PubMed, Web of Science, and Google Scholar. Quantitative, peer-reviewed empirical studies were included if they assessed maternal depression using standardized instruments and reported outcomes related to cognitive, language, motor, or socioemotional development in early childhood. Of 141 records identified, 10 studies met the eligibility criteria and were included in the qualitative synthesis.

**Results:**

The evidence indicates that maternal depression is not necessarily associated with global developmental delay, but rather with alterations in specific domains, including language, communication, problem solving, motor development, and socioemotional functioning. Symptom severity, persistence, and chronicity emerged as central predictors. Available studies suggested that the association may be partly explained by reduced maternal responsiveness, lower cognitive stimulation, difficulties in mother-child interaction, and adverse socioeconomic conditions.

**Conclusions:**

Maternal depression appears to be an important risk factor for child development during early childhood, particularly when symptoms are persistent or severe. The magnitude of these associations may vary according to caregiving environments and broader contextual factors.

## Introduction

Maternal depression represents a significant global public health concern, with important implications for maternal well-being and early child neurodevelopment [1]. The burden of maternal depressive symptoms is not evenly distributed across populations, with higher rates consistently reported in low- and middle-income countries and among socioeconomically vulnerable groups [2, 3, 4]. Furthermore, maternal depressive symptoms have been associated with less favorable developmental outcomes across multiple domains of child development [5, 6].

Current evidence suggests that exposure to postnatal depressive symptoms may be associated with less favorable outcomes across several domains of child neurodevelopment [5, 6]. However, contemporary research has shown that postpartum depression rarely causes global or generalized cognitive delay [7]. Instead, its associations tend to be domain-specific, particularly in receptive and expressive communication [8], as well as problem solving and fine motor skills [4]. Several prospective studies have reported associations between maternal depressive symptoms and less favorable outcomes in specific developmental domains, including communication, language, problem solving, and fine motor development [4, 8, 9]. Recent studies have also reported associations between maternal depression and less favorable executive function outcomes, including inhibitory control, cognitive flexibility, and attention from the preschool period onward [10].

Recent studies have used longitudinal symptom trajectories to examine how the persistence and severity of maternal depressive symptoms are associated with child developmental outcomes over time [4]. Studies suggest that the chronicity and severity of depressive symptoms are among the factors most consistently associated with developmental and behavioral difficulties in children [10, 11]. Indeed, even the chronicity of subclinical depressive symptoms—or persistently elevated depressive symptoms—has been associated with an increased risk of socioemotional and cognitive difficulties in children [10, 11].

One proposed mechanism linking maternal depression and child developmental outcomes involves alterations in mother-child interactions, infant social behavior, and reduced opportunities for cognitive stimulation within the caregiving environment [12, 13]. Maternal depressive symptoms have been associated with lower levels of sensitive responsiveness to infant needs and with less adaptive parenting behaviors, including withdrawn, lax, or overreactive patterns [3, 13]. Previous research has identified parenting behaviors as potential pathways linking maternal depressive symptoms with early child developmental outcomes [3]. Some intervention studies suggest that treating maternal depression while improving parenting practices may mitigate these adverse effects [14].

Despite the growing body of evidence on these potential pathways, notable discrepancies in the magnitude of reported effects suggest the presence of powerful socioenvironmental moderators [8, 13, 15]. Thus, in samples from families with high resources and comprehensive social safety nets, lower cognitive scores initially observed at 4 months of age may completely dissipate by 13 months if the depressive episode remits [15]. In marked contrast, in geographic or social environments characterized by poverty, relative deprivation, or low maternal education, vulnerability conditions may increase the likelihood of adverse developmental outcomes associated with maternal depression, resulting in more severe and persistent cognitive and language delays [8, 13].

Given the methodological complexity of evaluating development across these multiple contexts and the urgent need to clarify the factors that perpetuate risk, it is essential to systematically synthesize the recent evidence. The objective of this systematic review is to synthesize the available evidence on the association between maternal depression and child developmental outcomes during the first 36 months of life, with particular emphasis on global cognitive functioning, problem-solving, and language acquisition.

## Methods

### Study Design

This systematic review was designed and structured in accordance with the standardized guidelines and recommendations of the Preferred Reporting Items for Systematic reviews and Meta-Analyses (PRISMA) 2020 statement, with the aim of ensuring transparency and rigor in the identification, selection, evaluation, and synthesis of the included studies. The search, selection, and data extraction protocol was formulated in advance to rigorously assess the existing literature on the impact of maternal depression, particularly during the perinatal and postpartum periods, on cognitive development, neurodevelopment, and executive function during early childhood.

This review focused on empirical studies examining the association between maternal depressive symptoms and child developmental outcomes in children aged 0 to 36 months. Nevertheless, given that some longitudinal studies assess depressive exposure during the early years of life and report cognitive or executive function outcomes at slightly later stages, such studies were considered when they provided relevant evidence on the continuity of early effects of maternal depression. This decision allowed the integration of studies on early cognitive development, neurodevelopment, and executive function while preserving the central focus of the review on early childhood.

The protocol for this systematic review was registered in the International Prospective Register of Systematic Reviews (PROSPERO; registration number CRD420261425932; https://www.crd.york.ac.uk/PROSPERO/view/CRD420261425932). The methodological procedure was established before the synthesis of results and included the PRISMA 2020 guidelines, the definition of eligibility criteria using Population, Intervention/Exposure, Comparison, Outcomes, and Study design (PICOS), management of the study selection process in Rayyan, and a narrative synthesis strategy. Eligibility criteria were defined according to the PICOS framework. Regarding the population (P), studies were included if they involved samples of children from birth to 36 months of age, as well as studies describing the child population as infants, toddlers, or early childhood, provided that the developmental assessment age corresponded to early childhood or that the findings could be linked to early exposure to maternal depression.

Regarding exposure (I), studies were considered if they assessed maternal depression, maternal depressive symptoms, perinatal, prenatal, postnatal, or postpartum depression using standardized instruments, validated scales, or diagnostic interviews. Studies measuring depression at different time points were included: during pregnancy, in the early postpartum period, during the first months of the child’s life, or longitudinally through depressive symptom trajectories.

No specific comparator (C) was required, as many studies examined the association between maternal depression and child development using continuous variables, regression models, longitudinal analyses, trajectory models, or class/profile analyses. Studies comparing groups of mothers with and without depression, or groups defined by symptom severity, persistence, or chronicity, were also included.

Regarding outcomes (O), studies were included if they assessed cognitive development, neurodevelopment, language, communication, problem solving, motor development, personal-social development, socioemotional development, or executive function. Eligible studies used standardized or validated child development instruments, such as the Bayley Scales of Infant and Toddler Development, the Ages and Stages Questionnaire (ASQ), the Denver Developmental Screening Test II (DDST-II), executive function tasks, or other direct or reported measures of early development.

Regarding study design (S), quantitative empirical studies were included if they were cross-sectional, longitudinal, prospective, cohort-based, trajectory analyses, latent class analyses, or intervention studies, provided that they explicitly examined the relationship between maternal depression and child developmental outcomes. Systematic reviews, meta-analyses, narrative reviews, book chapters, theses, grey literature, and conference abstracts were excluded.

The literature search was conducted in scientific databases relevant to maternal mental health, child development, developmental psychology, pediatrics, and perinatal psychiatry. The main databases used were PsycINFO, SciELO, PubMed, Web of Science, and Google Scholar. The literature search was conducted between March and April 2026. Searches were complemented by manual review of the reference lists of included studies and related articles.

The search strategy combined terms related to maternal depression and early child development. The main terms used were: maternal depression, postpartum depression, postnatal depression, perinatal depression, prenatal depression, depressive symptoms, infant development, child development, cognitive development, neurodevelopment, executive function, language development, developmental delay, infant, toddler, and early childhood.

The general search string was adapted to the syntax of each database using Boolean operators. A base strategy was as follows: (“maternal depression” OR “postpartum depression” OR “postnatal depression” OR “perinatal depression” OR “prenatal depression” OR “depressive symptoms”) AND (“cognitive development” OR “neurodevelopment” OR “executive function” OR “child development” OR “infant development” OR “language development” OR “developmental delay”) AND (“infant” OR “toddler” OR “early childhood” OR “0–36 months”).

Articles published in English, Spanish, or Portuguese, available in full text and published in peer-reviewed journals, were included. The PRISMA 2020 checklist is provided as **Supplementary Material**.

Due to substantial methodological heterogeneity across the included studies, including differences in study design, maternal depression assessment, developmental outcome measures, follow-up periods, and statistical approaches, a quantitative synthesis (meta-analysis) was not considered appropriate. Therefore, the findings were integrated using a narrative synthesis.

### Study Selection Process

The identified records were organized and managed using Rayyan, a tool used to support the systematic review process, facilitate duplicate detection, and record inclusion and exclusion decisions. In the first stage, all retrieved records were imported into Rayyan, where duplicates were identified and removed.

Subsequently, titles and abstracts were screened using Rayyan by the first author and a co-author, excluding clearly irrelevant studies according to the predefined PICOS criteria. In the second stage, potentially eligible articles were reviewed in full text. During this phase, each study was assessed to determine whether it met the inclusion criteria related to population, exposure, outcomes, and methodological design. Eligibility decisions and uncertain cases were discussed among the authors until consensus was reached. Reasons for exclusion were systematically recorded, mainly including child age outside the defined range, absence of maternal depression measurement, absence of cognitive development/neurodevelopment or executive function assessment, non-eligible design, lack of empirical data, duplicate results, or preliminary publication replaced by a final published version.

The study selection process was summarized using a PRISMA 2020 flow diagram. A total of 141 records were identified through database searching. After removing 81 duplicate records, 60 records remained for title and abstract screening. Of these, 48 records were excluded for not meeting the inclusion criteria. Subsequently, 12 full-text articles were assessed for eligibility. Following full-text review, 2 studies were excluded because they assessed developmental outcomes beyond 36 months of age. Consequently, 10 studies met the eligibility criteria and were included in the qualitative synthesis of this systematic review (Fig. 1).

**Fig. 1.**
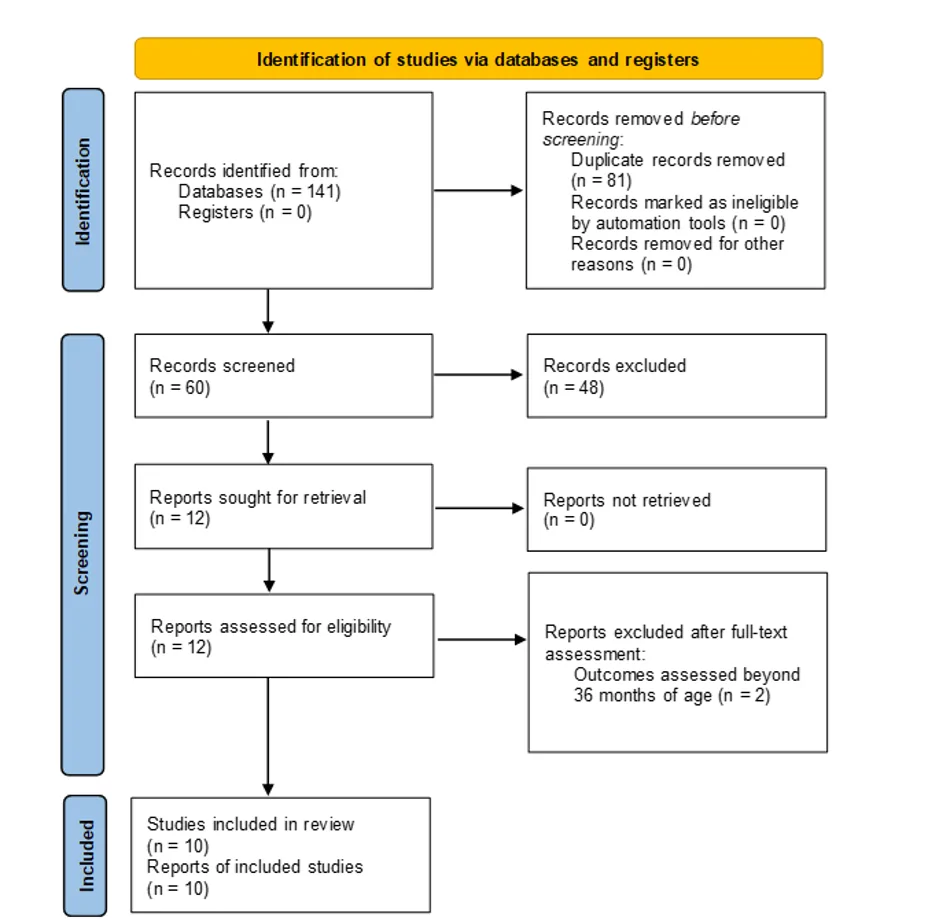
**PRISMA 2020 flow diagram of the study selection process**.

### Quality Assessment

The methodological quality of the included studies was assessed using the Joanna Briggs Institute (JBI) Critical Appraisal Checklists [16, 17]. For descriptive purposes in the present review, total methodological quality scores were calculated by summing the number of checklist items rated as “Yes”. Because the JBI checklists do not prescribe standardized cut-off scores for overall methodological quality, predefined thresholds were applied in this review solely to facilitate descriptive interpretation of the appraisal results. Cohort studies meeting 9–11 of the 11 criteria were categorized as having high methodological quality, whereas the study meeting 8 of the 11 criteria was categorized as having moderate methodological quality. A summary of the methodological quality assessment is presented in Table 1 (Ref. [2, 4, 5, 7, 8, 9, 12, 13, 14, 15, 16, 17]), and detailed item-level ratings, appraisal criteria (Q1–Q11 and Q1–Q13), and response categories are provided in **Supplementary Tables 1 and 2**.

**Table 1.** **Methodological quality assessment using the JBI critical appraisal checklist**.

Study	Design	JBI score	Quality
Ait Belkacem [5] (2024)	Cohort	11/11	High
De Oliveira [9] (2019)	Cohort	11/11	High
de Oliveira [2] (2025)	Cohort	11/11	High
Li [4] (2024)	Cohort	10/11	High
Parada [8] (2024)	Cohort	10/11	High
Schiavo [7] (2020)	Cohort	9/11	High
Smith-Nielsen [15] (2016)	Cohort	10/11	High
Smith-Nielsen [12] (2019)	Cohort	8/11	Moderate
Stein [14] (2018)	RCT	11/13	High
Viragova [13] (2025)	Cohort	9/11	High

Note: Cohort studies were assessed using the Joanna Briggs Institute (JBI) Critical Appraisal Checklist for Cohort Studies [16], whereas Stein *et al*. [14] (2018) was assessed using the JBI Critical Appraisal Checklist for Randomized Controlled Trials [17]. For descriptive purposes in the present review, total scores were calculated by summing the number of checklist items rated as “Yes”. Because the JBI checklists do not prescribe standardized cut-off scores for overall methodological quality, predefined thresholds were applied solely to facilitate descriptive interpretation. Cohort studies meeting 9–11 of the 11 criteria were categorized as having high methodological quality, whereas the study meeting 8 of the 11 criteria was categorized as having moderate methodological quality. Detailed item-level ratings, appraisal criteria (Q1–Q11/Q1–Q13), and response categories are provided in **Supplementary Tables 1 and 2**.

## Results

Overall, most included studies presented moderate to high methodological quality. Common strengths included longitudinal designs, the use of validated instruments, and relatively large cohort samples. The most frequent limitations were attrition during follow-up, reliance on maternal self-report measures, and heterogeneity in the control of confounding variables across studies.

Data extraction was conducted using an ad hoc matrix designed for this systematic review. For each of the 10 included studies, bibliographic, methodological, and substantive information relevant to the review objective was recorded.

Extracted variables included: author(s) and year of publication, country or context of the study, methodological design, sample size and characteristics, age of the children evaluated, timing of maternal depression assessment, instrument used to measure depressive symptoms, instrument used to assess child development, developmental domain evaluated, main findings, limitations reported by the authors, and relevance to the review question.

This matrix made it possible to compare the included studies according to their methodological characteristics, instruments used, timing of measurement, and main findings, facilitating an organized synthesis of the available evidence (Table 2, Ref. [2, 4, 5, 7, 8, 9, 12, 13, 14, 15]; Table 3).

**Table 2.** **Characteristics of the studies included in the systematic review**.

Author(s) and year	Design and sample (N)	Measures (depression/development)	Child age	Developmental domain	Key findings
Ait Belkacem [5] (2024)	Population-based cohort; N = 472	EPDS, GAD-7/ASQ-3	18 months	Communication, motor, problem solving	Severe prenatal psychological distress was associated with poorer communication and motor outcomes, whereas prenatal depressive symptoms showed an unexpected association with problem-solving outcomes.
De Oliveira [9] (2019)	Cohort; N = 1205 (Pakistan)	SRQ-20/Bayley-III	24 months	Cognitive, language, motor, socioemotional	Chronic maternal depressive symptoms were associated with poorer socioemotional development, whereas associations with cognitive, language, and motor development were no longer significant after covariate adjustment.
de Oliveira [2] (2025)	Prospective cohort; N = 840 (Brazil)	EPDS, GAD-7, IES/CDC milestones	24 months	Socioemotional, language, cognitive, motor	Increasing depressive symptom trajectories predicted a higher risk of developmental delays, particularly in language, cognitive, and motor domains.
Li [4] (2024)	Prospective cohort; N = 988 (China)	EPDS/ASQ-3, ASQ-SE	8 months	Communication, gross motor, fine motor, problem solving, personal-social	Persistent depressive symptom trajectories were associated with poorer fine motor, problem-solving, and socioemotional outcomes.
Parada [8] (2024)	Prospective cohort; N = 1555 (Brazil)	CES-D, EPDS/Bayley-III	12–24 months	Cognitive, language, motor	Maternal depression was associated with poorer receptive language, particularly in socioeconomically disadvantaged settings.
Schiavo [7] (2020)	Prospective cohort; N = 139 (Brazil)	BDI/DDST-II	6–14 months	Motor, language, personal-social	Depressive symptoms were associated with delays in specific developmental domains, but not with global developmental risk.
Smith-Nielsen [15] (2016)	Observational cohort; N = 83	EPDS, SCID, PSE/Bayley-III	4 and 13 months	Cognitive, language, motor	Lower cognitive scores were observed at 4 months among infants of depressed mothers, but these differences were no longer evident at 13 months.
Smith-Nielsen [12] (2019)	Cohort; N = 69	EPDS, PSE/Bayley-III, ADBB	4 months	Cognitive, social withdrawal	Maternal depressive symptoms were associated with lower cognitive scores; infant social withdrawal partially mediated this association.
Stein [14] (2018)	Randomized controlled trial; N = 144	EPDS, SCID/Bayley-III, CBCL, AQS	24 months	Cognitive, language, behavioral, attachment	No significant developmental differences were observed between maternal treatment conditions.
Viragova [13] (2025)	Longitudinal cohort; N = 479 (UK)	EPDS/Bayley-III, ECBQ, Q-CHAT, CBCL	~18 months	Neurocognitive, affective regulation	Associations between maternal depression and developmental outcomes were mediated by reduced cognitive stimulation and maladaptive parenting behaviors.

EPDS, Edinburgh Postnatal Depression Scale; GAD-7, Generalized Anxiety Disorder-7; ASQ-3, Ages and Stages Questionnaire, Third Edition; SRQ-20, Self-Reporting Questionnaire-20; Bayley-III, Bayley Scales of Infant and Toddler Development, Third Edition; IES, Impact of Event Scale; CDC, Centers for Disease Control and Prevention; ASQ-SE, Ages and Stages Questionnaire: Social-Emotional; CES-D, Center for Epidemiologic Studies Depression Scale; BDI, Beck Depression Inventory; DDST-II, Denver Developmental Screening Test II; SCID, Structured Clinical Interview for DSM Disorders; PSE, Present State Examination; ADBB, Alarm Distress Baby Scale; CBCL, Child Behavior Checklist; AQS, Attachment Q-Sort; ECBQ, Early Childhood Behavior Questionnaire; Q-CHAT, Quantitative Checklist for Autism in Toddlers.

**Table 3.** **Geographic distribution of included studies**.

Continent	Country	n	% of total
Europe	United Kingdom	2	20%
	Denmark	2	20%
South America	Brazil	3	30%
North America	Canada	1	10%
Asia	China	1	10%
	Pakistan	1	10%
Total		10	100%

### Instruments Used to Measure Maternal Depression

To evaluate the severity, chronicity, and trajectory of maternal depressive symptomatology, the studies included in this review used multiple standardized instruments. As detailed in Table 4 (Ref. [2, 4, 5, 7, 8, 9, 12, 13, 14, 15]), the Edinburgh Postnatal Depression Scale (EPDS) emerged as the predominant tool, being used as the main screening method in eight studies [2, 4, 5, 8, 12, 13, 14, 15]. One study used the Center for Epidemiologic Studies Depression Scale (CES-D) [8], another used the Beck Depression Inventory (BDI) [7], and one study employed the Self-Reporting Questionnaire (SRQ-20) [9]. Methodologically, three studies complemented self-report questionnaires with structured psychiatric interviews. Specifically, Smith-Nielsen *et al*. [15] (2016) used both the Structured Clinical Interview for DSM Disorders (SCID) and the Present State Examination (PSE), Smith-Nielsen *et al*. [12] (2019) used the PSE, and Stein *et al*. [14] (2018) used the SCID, thereby strengthening diagnostic validity beyond self-report measures.

**Table 4.** **Instruments used to measure maternal depression**.

Measurement instrument	Studies that used it	Count
EPDS	Parada [8] (2024); Smith-Nielsen [12] (2019); Li [4] (2024); Smith-Nielsen [15] (2016); Viragova [13] (2025); Ait Belkacem [5] (2024); Stein [14] (2018); de Oliveira [2] (2025)	8
CES-D	Parada [8] (2024)	1
SCID	Smith-Nielsen [15] (2016); Stein [14] (2018)	2
PSE	Smith-Nielsen [12] (2019); Smith-Nielsen [15] (2016)	2
BDI	Schiavo [7] (2020)	1
SRQ-20	De Oliveira [9] (2019)	1

Methodological note: EPDS, Edinburgh Postnatal Depression Scale; CES-D, Center for Epidemiologic Studies Depression Scale; SCID, Structured Clinical Interview for DSM Disorders; PSE, Present State Examination; BDI, Beck Depression Inventory; SRQ-20, Self-Reporting Questionnaire. The sum of the “Count” column exceeds the number of included studies (N = 10) because some studies used more than one instrument to assess maternal depressive symptomatology or combined self-report measures with structured clinical interviews. The EPDS was the most frequently used instrument, appearing in eight of the ten included studies.

Europe (40%): This continent had the greatest representation in the final selection. It included two studies from the United Kingdom [13, 14] and two observational studies conducted in Denmark [12, 15].

South America/Brazil (30%): Brazil was the individual country contributing the largest number of studies to the review, with three longitudinal studies conducted in different socioeconomic contexts [2, 7, 8].

North America (10%): This region was represented by one Canadian cohort, the CONCEPTION study, which examined the relationship between maternal mental health during pregnancy and child cognitive development [5].

Asia (20%): This region included a prospective cohort conducted in Shenzhen, China, focused on maternal depressive symptom trajectories [4], and a cohort in rural Pakistan evaluating the effects of chronic maternal depressive symptoms on child development [9].

### Measures of Cognitive Development

To evaluate child developmental outcomes, the studies included in this review used a variety of standardized assessment instruments. As detailed in Table 5 (Ref. [2, 4, 5, 7, 8, 9, 12, 13, 14, 15]), the Bayley Scales of Infant and Toddler Development, Third Edition (Bayley-III), emerged as the most frequently used measure, being applied in six studies [8, 9, 12, 13, 14, 15]. For population-based studies and developmental screening, the Ages and Stages Questionnaire, Third Edition (ASQ-3), was used in two studies [4, 5]. Other studies employed the DDST-II [7] and the Centers for Disease Control and Prevention (CDC) developmental milestones checklist [2]. Together, these instruments enabled the assessment of multiple developmental domains, including cognitive, language, motor, and socioemotional development.

**Table 5.** **Instruments used to measure child cognitive development**.

Measurement instrument	Studies that used it	Count
Bayley-III	Parada [8] (2024); Smith-Nielsen [12] (2019); Smith-Nielsen [15] (2016); Viragova [13] (2025); Stein [14] (2018); De Oliveira [9] (2019)	6
ASQ-3	Li [4] (2024); Ait Belkacem [5] (2024)	2
DDST-II	Schiavo [7] (2020)	1
CDC developmental milestones	de Oliveira [2] (2025)	1

Methodological note: Bayley-III, Bayley Scales of Infant and Toddler Development, Third Edition; ASQ-3, Ages and Stages Questionnaire, Third Edition; DDST-II, Denver Developmental Screening Test II; CDC developmental milestones, Centers for Disease Control and Prevention developmental milestones checklist. The sum of the “Count” column equals the number of included studies (N = 10), as each study was classified according to its primary developmental assessment instrument.

### Results by Developmental Domain

#### Cognitive Development

Seven studies examined cognitive development in relation to maternal depressive symptoms [2, 8, 9, 12, 13, 14, 15]. Overall, the evidence suggests that maternal depressive symptoms may be associated with less favorable cognitive outcomes; however, these associations were not consistently observed across all studies. Persistent or increasing depressive symptom trajectories were more frequently associated with poorer cognitive performance and delayed developmental milestones than transient symptoms [2, 9]. Likewise, lower cognitive scores were reported during early infancy among children of mothers with clinical depression, although these differences were no longer evident following maternal symptom remission [15]. Maternal depressive symptoms were also associated with lower cognitive performance during infancy [12], whereas indirect pathways involving reduced cognitive stimulation and parenting behaviors were proposed as potential explanations for these associations [13]. In contrast, one randomized controlled trial found no significant differences in cognitive outcomes between maternal treatment groups at 24 months [14].

#### Language and Communication Development

Language and communication emerged as one of the developmental domains most consistently associated with maternal depressive symptoms. Eight studies assessed language and/or communication outcomes [2, 4, 5, 7, 8, 9, 14, 15]. Across the included studies, persistent or increasing depressive symptoms were associated with poorer receptive communication, delayed language milestones, and lower communication scores during infancy and toddlerhood [2, 4, 8]. Severe prenatal psychological distress was also associated with poorer communication outcomes at 18 months [5]. However, one study reported that the association between maternal depression and receptive communication was evident only in the socioeconomically disadvantaged setting, suggesting that contextual factors may influence these associations [8].

#### Motor Development

Seven studies evaluated motor development [2, 4, 5, 7, 8, 9, 15]. Maternal depressive symptoms were associated with less favorable gross motor, fine motor, or overall motor outcomes in several studies [2, 4, 5, 7]. However, these associations were not consistently observed. In De Oliveira *et al*. [9], associations with motor development were no longer significant after covariate adjustment, whereas Smith-Nielsen *et al*. [15] did not report persistent motor differences at the later assessment. Overall, the findings suggest that associations with motor development may vary according to symptom trajectory, assessment timing, and contextual factors.

#### Socioemotional and Personal-Social Development

Seven studies assessed socioemotional, personal-social, behavioral, attachment, social withdrawal, or affective outcomes [2, 4, 7, 9, 12, 13, 14]. Chronic or recurrent maternal depressive symptoms were consistently associated with poorer socioemotional development during the first two years of life [9]. Persistent depressive symptom trajectories were also associated with less favorable personal-social development [4], whereas maternal depressive symptoms were related to greater infant social withdrawal during early infancy [12]. In addition, delays in personal-social development were reported among infants whose mothers experienced postpartum depressive symptoms [7].

#### Mediating and Moderating Factors

Three studies formally examined factors that could explain variability in the association between maternal depression and child developmental outcomes [8, 12, 13]. Infant social withdrawal partially mediated the association between maternal depressive symptoms and cognitive development [12]. Reduced cognitive stimulation and parenting behaviors characterized by laxness or overactivity were also identified as indirect pathways linking maternal depressive symptoms to child developmental outcomes [13]. Finally, socioeconomic context appeared to moderate these associations, as poorer developmental outcomes were observed primarily in settings characterized by greater social disadvantage [8].

## Discussion

The findings suggest that maternal depressive symptoms may be associated with less favorable developmental outcomes; however, these associations were not uniform across developmental domains or study populations. Rather than indicating a generalized developmental delay, the included studies more consistently reported associations with specific domains, particularly cognitive development, language and communication, motor development, and socioemotional functioning [2, 4, 7, 8, 9, 12, 13, 15].

One of the most consistent findings across the included studies was the importance of symptom persistence and chronicity. Persistent or increasing depressive symptom trajectories were more frequently associated with less favorable developmental outcomes than transient symptoms [2, 4, 9]. In contrast, one longitudinal study found that lower cognitive scores observed during early infancy were no longer evident after maternal symptom remission, suggesting that developmental differences may diminish over time under favorable caregiving conditions [15]. Similarly, the randomized controlled trial conducted by Stein *et al*. [14] found no significant differences in child cognitive outcomes between maternal treatment groups at 24 months. Collectively, these findings indicate that symptom persistence, rather than the mere presence of maternal depressive symptoms, may be more relevant for child developmental outcomes, although the caregiving context also appears to influence these associations [2, 4, 9, 13, 15].

The findings also suggest that maternal depression may influence child developmental outcomes indirectly through alterations in the caregiving environment. However, only two of the included studies formally evaluated these mechanisms using mediation analyses [12, 13]. Smith-Nielsen *et al*. [12] reported that infant social withdrawal partially mediated the association between maternal depressive symptoms and cognitive development, whereas Viragova *et al*. [13] identified reduced cognitive stimulation and less adaptive parenting behaviors as significant indirect pathways. The remaining studies discussed caregiving quality, maternal responsiveness, and environmental stimulation as plausible explanatory mechanisms but did not formally test these pathways. Therefore, these factors should be interpreted as proposed mechanisms rather than established causal pathways.

Another important finding concerns the influence of contextual factors. Studies conducted in socioeconomically disadvantaged settings generally reported stronger associations between maternal depressive symptoms and child developmental outcomes than those conducted in more favorable environments [8, 9]. Conversely, higher maternal education, greater social support, and more stimulating caregiving environments were consistently discussed as factors that may reduce developmental vulnerability [8, 13, 15]. These findings support the importance of considering maternal depression within a broader ecological framework in which clinical, relational, and socioeconomic factors interact [8, 13, 15].

The findings of this review should be interpreted considering several methodological limitations within the available evidence. Although most studies employed longitudinal designs and validated assessment instruments, substantial heterogeneity was observed regarding depression measures, developmental assessments, follow-up periods, and statistical approaches. In addition, relatively few studies directly examined mediating or moderating mechanisms, limiting conclusions regarding the processes underlying the observed associations. These methodological differences also precluded quantitative synthesis.

Overall, the available evidence suggests that maternal depression should be considered an important factor associated with early child development, particularly when symptoms are persistent or severe. At the same time, the variability observed across studies indicates that developmental outcomes are likely influenced by multiple interacting factors rather than maternal depressive symptoms alone [8, 9, 13, 15]. Future research should continue to investigate symptom trajectories, caregiving processes, and contextual conditions in order to better identify the mechanisms that increase or reduce developmental vulnerability.

### Limitations

This review should be interpreted considering some methodological considerations. First, the included studies varied in terms of sample characteristics, timing of maternal depression assessment, child developmental outcomes, and statistical approaches. Due to this substantial methodological heterogeneity, a quantitative synthesis (meta-analysis) was not considered appropriate, and the findings were therefore synthesized narratively. However, this heterogeneity also allowed for a broader understanding of the association between maternal depression and child development across different contexts. Second, although longitudinal designs predominated, follow-up duration and attrition rates varied across studies. Finally, the studies used different instruments to assess both maternal depressive symptoms and child cognitive development, which should be considered when interpreting the findings in an integrated manner.

## Conclusions

Maternal depression appears to be an important developmental risk factor during early childhood, particularly when symptoms are persistent or severe. Across the studies included, maternal depressive symptoms were most consistently associated with less favorable cognitive, language, motor, and socioemotional outcomes. However, the strength and consistency of these associations varied according to symptom trajectories, caregiving environments, and broader socioeconomic contexts. Furthermore, some studies reported domain-specific effects, while others found limited or no significant associations. These findings highlight the importance of considering maternal mental health within a broader ecological framework and support the need for early identification and intervention strategies. Future research should continue to examine the mechanisms underlying these associations and the contextual factors that may increase or reduce developmental vulnerability.

## Availability of Data and Materials

The datasets generated and/or analyzed during this research are not publicly available due to privacy and ethical restrictions, but are available from the corresponding author on reasonable request, subject to approval by respective Ethics Committee.

## References

[1] Trussell TM, Ward WL, Conners Edge NA (2018). The impact of maternal depression on children: a call for maternal depression screening. *Clinical pediatrics*.

[2] de Oliveira CVR, Bordignon SS, Martins RC, Blumenberg C, Martins-Silva T, Costa F (2025). Trajectories of maternal depression, anxiety, stress, and child developmental milestones at 24 months. *Journal of child and adolescent mental health*.

[3] Frost A, Scherer E, Chung EO, Gallis JA, Sanborn K, Zhou Y (2025). Longitudinal pathways between maternal depression, parenting behaviors, and early childhood development: a mediation analysis. *International journal of behavioral development*.

[4] Li X, Liu X, Wei M, Liu X, Shi X, Zhu Y (2024). Associations between maternal depression trajectories and infant neurodevelopment at eight months. *Early human development*.

[5] Ait Belkacem N, Gorgui J, Tchuente V, Aubin D, Lippé S, Bérard A (2024). Maternal Mental Health in Pregnancy and Its Impact on Children’s Cognitive Development at 18 Months, during the COVID-19 Pandemic (CONCEPTION Study). *Journal of clinical medicine*.

[6] Araújo ACC, Alves EIR, Araújo JAS, Oliveira MF, Mendes NVS, Duarte PLS (2024). O impacto da depressão pós-parto no desenvolvimento cognitivo infantil. *Journal of Health & Biological Sciences*.

[7] Schiavo RA, Perosa GB (2020). Child Development, Maternal Depression and Associated Factors: A Longitudinal Study. *Paidéia (Ribeirão Preto)*.

[8] Parada AP, de Rezende MG, de Paula Eduardo JAF, de Figueiredo FP, Bettiol H, Cavalli R (2024). Associações entre depressão materna e o desenvolvimento neurocomportamental das crianças: um estudo longitudinal e descritivo. *Prometeica*.

[9] De Oliveira CVR, Rasheed M, Yousafzai AK (2019). Chronic Maternal Depressive Symptoms Are Associated with Reduced Socio-Emotional Development in Children at 2 Years of Age: Analysis of Data From an Intervention Cohort in Rural Pakistan. *Frontiers in psychiatry*.

[10] Power J, Watson S, Chen W, Lewis A, van IJzendoorn M, Galbally M (2023). The trajectory of maternal perinatal depressive symptoms predicts executive function in early childhood. *Psychological medicine*.

[11] Netsi E, Pearson RM, Murray L, Cooper P, Craske MG, Stein A (2018). Association of Persistent and Severe Postnatal Depression with Child Outcomes. *JAMA psychiatry*.

[12] Smith-Nielsen J, Lange T, Wendelboe KI, von Wowern RK, Væver MS (2019). Associations Between Maternal Postpartum Depression, Infant Social Behavior with a Stranger, and Infant Cognitive Development. *Infancy*.

[13] Viragova M, Falconer S, Chew A, Edwards AD, Dazzan P, Nosarti C (2025). Environmental variables influence the relationship between maternal depressive symptoms and toddlers’ neurocognitive and affective outcomes. *Journal of affective disorders*.

[14] Stein A, Netsi E, Lawrence PJ, Granger C, Kempton C, Craske MG (2018). Mitigating the effect of persistent postnatal depression on child outcomes through an intervention to treat depression and improve parenting: a randomised controlled trial. The lancet. *Psychiatry*.

[15] Smith-Nielsen J, Tharner A, Krogh MT, Væver MS (2016). Effects of maternal postpartum depression in a well-resourced sample: Early concurrent and long-term effects on infant cognitive, language, and motor development. *Scandinavian journal of psychology*.

[16] Barker TH, Hasanoff S, Aromataris E, Stone JC, Leonardi-Bee J, Sears K (2025). The revised JBI critical appraisal tool for the assessment of risk of bias for cohort studies. *JBI evidence synthesis*.

[17] Barker TH, Stone JC, Sears K, Klugar M, Tufanaru C, Leonardi-Bee J (2023). The revised JBI critical appraisal tool for the assessment of risk of bias for randomized controlled trials. *JBI evidence synthesis*.

