# Supplementary material for: Maternal Depression and Its Relationship With Cognitive Development in Children Aged 0 to 36 Months: A Systematic Review
Source: Actas Esp Psiquiatr. 2026 Oct 15;54(5):1688–98. doi: 10.62641/aep.2339 (PMC13714477; doi:10.62641/aep.2339)
Supplement: Supplementary file 1 [file ActEsp-54-5-1688-1698-s1.zip › AEP-2339-Suplemmentary tables Final.docx]

**Table 1. Methodological quality assessment of included studies using the JBI Critical Appraisal Checklist for Cohort Studies**

| **Study** | **Q1** | **Q2** | **Q3** | **Q4** | **Q5** | **Q6** | **Q7** | **Q8** | **Q9** | **Q10** | **Q11** | **Total** |
| --- | --- | --- | --- | --- | --- | --- | --- | --- | --- | --- | --- | --- |
| Ait Belkacem (2024) | Y | Y | Y | Y | Y | Y | Y | Y | Y | Y | Y | 11/11 |
| De Oliveira (2019) | Y | Y | Y | Y | Y | Y | Y | Y | Y | Y | Y | 11/11 |
| De Oliveira (2025) | Y | Y | Y | Y | Y | Y | Y | Y | Y | Y | Y | 11/11 |
| Li (2024) | Y | Y | Y | Y | Y | Y | Y | Y | Y | N | Y | 10/11 |
| Parada (2024) | Y | Y | Y | Y | Y | Y | Y | Y | Y | N | Y | 10/11 |
| Schiavo (2020) | Y | Y | Y | Y | P | Y | P | Y | P | N | Y | 9/11 |
| Smith-Nielsen (2016) | Y | Y | Y | Y | Y | Y | Y | Y | Y | P | Y | 10/11 |
| Smith-Nielsen (2019) | Y | Y | Y | Y | Y | P | Y | P | P | P | Y | 8/11 |
| Virágová (2025) | Y | Y | Y | Y | Y | Y | Y | Y | P | P | Y | 9/11 |

**Note**: Y = Yes; N = No; U = Unclear; NA = Not applicable; P = Partially met.

**BI Critical Appraisal Checklist for Cohort Studies**

**Q1**. Were the two groups similar and recruited from the same population?

**Q2**. Were the exposures measured similarly to assign people to both exposed and unexposed groups?

**Q3**. Was the exposure measured in a valid and reliable way?

**Q4**. Were confounding factors identified?

**Q5**. Were strategies to deal with confounding factors stated?

**Q6**. Were the groups/participants free of the outcome at the start of the study?

**Q7**. Were the outcomes measured in a valid and reliable way?

**Q8**. Was the follow-up time reported and sufficient?

**Q9**. Was follow-up complete, and if not, were reasons described?

**Q10**. Were strategies to address incomplete follow-up utilized?

**Q11**. Was appropriate statistical analysis used?

**Table 2. Methodological quality assessment of included studies using the JBI Critical Appraisal Checklist for Randomized Controlled Trials**

| **Study** | **Q1** | **Q2** | **Q3** | **Q4** | **Q5** | **Q6** | **Q7** | **Q8** | **Q9** | **Q10** | **Q11** | **Q12** | **Q13** | **Total** |
| --- | --- | --- | --- | --- | --- | --- | --- | --- | --- | --- | --- | --- | --- | --- |
| Stein (2018) | Y | Y | Y | N | N | Y | Y | Y | Y | Y | Y | Y | Y | **11/13** |

**Note**: Y = Yes; N = No; U = Unclear; NA = Not applicable; P = Partially met.

**JBI Critical Appraisal Checklist for Randomized Controlled Trials**

**Q1.** Was true randomization used for assignment of participants to treatment groups?

**Q2.** Was allocation to treatment groups concealed?

**Q3.** Were treatment groups similar at baseline?

**Q4.** Were participants blind to treatment assignment?

**Q5.** Were those delivering treatment blind to treatment assignment?

**Q6.** Were outcome assessors blind to treatment assignment?

**Q7.** Were treatment groups treated identically other than the intervention of interest?

**Q8.** Was follow-up complete, and if not, were differences adequately described and analyzed?

**Q9.** Were participants analyzed in the groups to which they were randomized?

**Q10.** Were outcomes measured in the same way for treatment groups?

**Q11.** Were outcomes measured in a reliable way?

**Q12.** Was appropriate statistical analysis used?

**Q13.** Was the trial design appropriate, and were any deviations from the standard RCT design accounted for?
